# Meningococcal carriage among Hajj pilgrims, risk factors for carriage and records of vaccination: a study of pilgrims to Mecca

**DOI:** 10.1111/tmi.13546

**Published:** 2021-01-31

**Authors:** Abrar Alasmari, Joanna Houghton, Brian Greenwood, David Heymann, Phil Edwards, Heidi Larson, Abdullah Assiri, Fathia Ben‐Rached, Arnab Pain, Ron Behrens, Amaya Bustinduy

**Affiliations:** ^1^ Department of Clinical Research London School of Hygiene & Tropical Medicine London UK; ^2^ Department of Disease Control London School of Hygiene & Tropical Medicine London UK; ^3^ Chatham House Centre on Global Health Security London UK; ^4^ Department of Infectious Disease Epidemiology London School of Hygiene & Tropical Medicine London UK; ^5^ Department of Population Health London School of Hygiene & Tropical Medicine London UK; ^6^ Department of Global Health Institute for Health Metrics and Evaluation University of Washington Seattle WA USA; ^7^ Preventive Health Ministry of Health Riyadh Saudi Arabia; ^8^ Biological and Environmental Sciences and Engineering Division King Abdullah University of Science and Technology (KAUST) Thuwal‐Jeddah Saudi Arabia; ^9^ Global Institution for Collaborative Research and Education Research Center for Zoonosis Control Hokkaido University Sapporo Japan

**Keywords:** antibiotic, Hajj, meningococcal, *Neisseria meningitides*, pharyngeal carriage, vaccination

## Abstract

**Objective:**

The Saudi government requires that all pilgrims receive a quadrivalent meningococcal vaccine at least 10 days before the Hajj. We conducted a study to determine the uptake of meningococcal vaccine and antibiotic use. We also investigated risk factors of meningococcal carriage and carriage of *Neisseria meningitidis* pathogenic serogroups A, C, W and Y.

**Methods:**

A cross‐sectional oropharyngeal carriage survey was conducted in 2973 Hajj pilgrims in September 2017. A real‐time polymerase chain reaction (rt‐PCR) assay was used to identify *N. meningitidis* from the oropharyngeal swabs. A questionnaire investigated potential risk factors for carriage of *N. meningitidis*.

**Results:**

Two thousand two hundred forty nine oropharyngeal swabs were obtained. The overall prevalence of carriage of *N. meningitidis* was 4.6% (95% CI: 3.4%–6%). Carriage of pathogenic serogroups was not associated significantly with any of the meningococcal risk factors evaluated. 77% of pilgrims were vaccinated but 22.58 % said they were carrying unofficial vaccination cards.

**Conclusion:**

Carriage of serogroups A, C, W and Y was not significantly associated with any of the risk factors investigated. Almost a quarter of pilgrims were unlikely to have been vaccinated, highlighting a need to strengthen compliance with the current policy of vaccination to prevent meningococcal disease outbreaks during and after the Hajj.

## Introduction

*Neisseria meningitidis* is a gram‐negative aerobic bacterium which causes invasive meningococcal disease, a communicable disease spread via respiratory droplets [1]. There are twelve identified serogroups of *N. meningitidis*, which can be distinguished from each other by their polysaccharide capsule. However, there are six serogroups (A, B, C, W135, X and Y) that cause invasive disease [2]. Meningococcal carriers who have bacteria in the oropharynx but do not present any symptoms are the main source of invasive infections [1, 3].

Over two million Muslims visit Mecca in Saudi Arabia every year to perform the Hajj, one of the largest mass gatherings in the world [4]. Pilgrims typically share accommodation with other pilgrims and stay in tents during the 5 days of Hajj season [5]. Overcrowding during the Hajj and an increase in the number of pilgrims inside Hajj tents has, in the past, facilitated the spread of meningococcal disease, and there have been several meningococcal outbreaks during Hajj pilgrimage [4]. As a consequence of these outbreaks, the Saudi authorities developed and upgraded their Hajj vaccination policy to mandatory quadrivalent meningococcal vaccination, which must be administered to all Hajj pilgrims before arriving in Saudi Arabia [6]. A vaccine that protects against all serogroups is currently unavailable [7]. The existing Hajj vaccination policy does not indicate the type of quadrivalent (ACYW135) vaccine that should be administered, that is, a quadrivalent meningococcal conjugate vaccine (MCV‐4) or a quadrivalent meningococcal polysaccharide vaccine (MPSV‐4) [8]. Meningococcal polysaccharide vaccination can prevent severe meningococcal illnesses, but it does not prevent the acquisition of carriage [1]. A quadrivalent conjugate vaccine may prevent acquisition of new carriage, but it does not clear existing carriage which may take 2 months or more to clear naturally [9]. A quadrivalent meningococcal ACWY glycoconjugate vaccine was shown to have little impact on carriage 1 month post vaccination [9]. This may have an impact on Hajj vaccination policy, which currently only requires vaccination 10 days prior to travelling to the Hajj [10].

Self‐medicating with antibiotics has long been customary among pilgrims during the Hajj period to protect themselves against diseases transmitted via the respiratory route [11]. This has most likely played a role in eliminating carriage in previously reported Hajj studies [12, 13]. Conversely, this custom of administration of non‐prescribed antibiotics by Hajj pilgrims may contribute to increasing antibiotic resistance [11].

The aim of this study was to determine the uptake of the meningococcal vaccine and the use of antibiotics by Hajj pilgrims. The study also aimed to investigate the rate of *N. meningitidis* carriage among pilgrims and to determine the risk factors associated with the carriage of *N. meningitidis* serogroups (A, C, W and Y).

## Methods

### Study design and setting

A cross‐sectional study was conducted in Jeddah, Saudi Arabia, at the Hajj terminal of King Abdulaziz International Airport (KAIA) after the 2017 Hajj. Most of the international pilgrims pass through the Hajj terminal when visiting the Sacred Mosque in Mecca.[14]

### Sampling methods

Two stage cluster sampling was used in the Hajj terminal at KAIA to select participants for the study. Departing flights from the Hajj terminal were selected as clusters as the first stage using simple random sampling from the daily Hajj flight schedules. Subsequently, at the second stage, systematic random sampling of each flight cluster was undertaken using seat numbering. Departing pilgrims were recruited in the airport lounge, and only those who provided informed consent were included in the study.

### Data collection

All pilgrims selected for inclusion in the study were provided with written information regarding the aims of the study. Upon their agreement to participate, they were asked to sign a consent form. An electronic data capture tool, ‘Open Data Kit’ (ODK) was used to collect questionnaire data [15]. Assistance was provided to pilgrims with clarifying questions and on how to use electric tablets.

### Questionnaire design, piloting and translation

Twenty electronic tablets (Asus Zenpad 8 Z580C) supported by designed data questionnaire forms for 15 languages including Arabic, Albanian, Bengali, Bosnian, Chinese, English, French, Hindi, Indonesian, Kurdish, Malay, Pashto, Russian, Turkish and Urdu were used to collect data from pilgrims. All translated questionnaire forms were piloted prior to conducting the study.

### Samples collection and storage

A Dacron/polyester tip swab was gently rolled over the tonsils and posterior pharynx and inserted into a vial containing transport medium containing skimmed milk, tryptone, glucose and glycerine (STGG). All swabs were kept at 4°C in an ice box for 1 h followed by storage in a portable freezer at −20°C. They were then kept securely at the airport for 2 weeks prior to being transported to King Abdullah University of Science and Technology (KAUST) where they were stored at −80°C and then shipped on a dry ice to London School of Hygiene and Tropical Medicine for laboratory investigations. No cold chain breakdown was recorded during shipment.

### Laboratory analysis

An aliquot of 300 μl STGG medium was extracted from each vial and purified using QIAamp cador Pathogen Mini Kit (Cat No. /ID: 54106), following the manufacturer’s protocol. Extracted DNA was eluted in 100 μl of elution buffer and stored at −20°C. Quantitative PCR was then used to detect *N. meningitidis ctrA*, *sodC* and *porA* target genes separately using primers and probes as described previously [16, 17]. It is recommended to use a duplex real‐time PCR approach that targets both *porA* and *ctrA* genes as these two are effective in detecting most *N. meningitidis* invasive strains. Non‐groupable (NG) strains can also be identified by using *sodC* assay, which is superior to *ctrA* assay in detecting NG strains of *N. meningitides* [18].

All positive samples were tested for capsular biosynthesis genes for *N. meningitidis* serogroups A, B, C, W, X and Y using primers and probes as described previously [17]. All PCR reactions were performed with 5 µl of extracted DNA and 10 µl of qPCRBIO Probe mix Hi‐ROX (PCR Biosystems PB20·22) in a reaction volume of 20 µl using the 7500 ABI platform (Applied Biosystem). Samples were tested in duplicates and considered as positive when the sample had a cycle threshold (Ct) value below 40 [19]. All samples were tested in parallel with positive controls for each serogroup (kindly supplied by Dr Odile Harrison, University of Oxford).

### Statistical analysis

A descriptive analysis of all survey variables was performed using Stata commands for survey data (SVY) to account for the multistage sampling design. All analyses were weighted for probability of selection. Flight numbers and number of pilgrims in each flight were used to calculate the weight used in the analysis to ensure that the probability of selection for each pilgrim sampled from flight was the same as the overall probability of selection for all pilgrims. A logistic regression model was developed to examine the associations between risk factors for meningococcal carriage (age, sex, education, type of meningococcal vaccine, timing of meningococcal vaccination, smoking status, marital status, country classification by income, length of stay in Saudi Arabia and number of pilgrims inside the tent where the participant slept) and the binary outcome variable ‘meningococcal carriage of serogroup ACWY’. Vaccination time was calculated from the date of receiving vaccination until the Hajj dates and was categorised as 0: ≤ 60 days, 1: ≥ 61 days. It is suggested that 2 months are needed to naturally clear any existing carriage of *N. meningitidis,* and therefore, 2 months were used as a cut‐off point [10]. The Wald test was used to assess evidence for any associations between the outcome ‘meningococcal carriage of serogroup ACWY’ and each of the potential variables mentioned above. Due to lack of observations in some categories of variables, variables such as age, education and country classification by income were re‐categorised to fit the regression model. Age was categorised as 0: ≤ 34 years, 1: 35–44 years, 2: 45–54 years, 3: 55–64 years and 4: ≥ 65 years. Education was classified as 0: low (Illiterate pilgrims or those who could only read and write), 1: middle (pilgrims with qualification of 2 years college, high school or less than high school) and 2: high (pilgrims with doctoral, master's or bachelor's degree). The type of meningococcal vaccine was categorised as 0: bivalent A and C or quadrivalent polysaccharide, 1: quadrivalent conjugate and 2: unknown type. Country classification by income, as defined by the World Bank report for 2017–2018 [20], was also categorised as 0: low and lower middle‐income countries, 1: upper middle‐income countries and 2: high‐income countries. Variables with a *P* > 0.1 were considered statistically insignificant. All analyses were conducted using STATA 16 software [21].

### Ethical approval

Ethical approvals were obtained from the ethics committees of the London School of Hygiene and Tropical Medicine and King Abdullah University of Science and Technology prior to conducting the research.

## Results

### Demographic and other baseline characteristics

Of the initial 2973 participants, 2249 (75.56%) completed the electronic questionnaire and agreed to be swabbed. Table 1 summarises pilgrims' demographic data. Participants came from China, Europe, East Africa, the Middle East, North Africa, North America, Post‐soviet states, South Asia, Southeast Asia, West Africa and South Africa. The total length of stay of pilgrims inside Saudi Arabia during the Hajj journey (*n* = 2973) averaged 33.8 (SD: 12.92) days in the 2017 Hajj season.

**Table 1:** Demographic characteristics of pilgrims

**Characteristics**	Total (%)
Sex	
Female	32
Male	68
Age in years	
11–17	0.5
18–24	2.9
25–34	12.6
35–44	26.7
45–54	26.3
55–64	22.5
65 or above	8.5
Education level	
Illiterate	5
Can read and write	11
Less than high school	9
High school	19
Two years college	9
Bachelor degree	27.5
Master's degree	15
Doctoral degree	4.5
Country classification by income	
Low	6
Lower‐middle	57
Upper‐middle	28
High	9
Marital status	
Married	87
Unmarried	13

### Meningococcal vaccination status and antibiotic use

**Figure 1:**
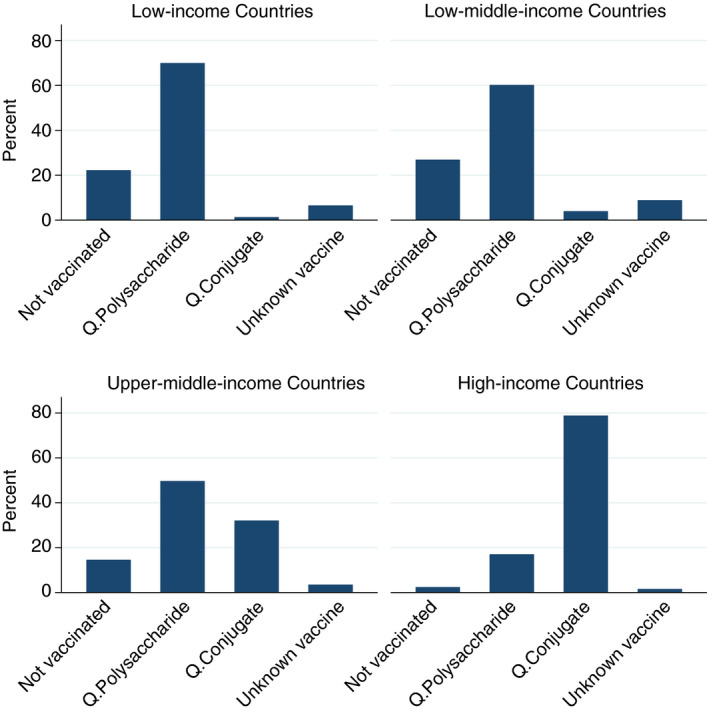
Vaccination status of pilgrim by country classification by income.

The survey showed that 22.6 % of study participants stated that they had not been vaccinated against meningococcal disease; 12.5 % of those were not vaccinated and not carrying any vaccination certificates, and 11% self‐reported that they were unvaccinated and carrying an unofficial purchased vaccination certificate. Very few pilgrims (0.5 %) had received the bivalent polysaccharide (A and C) meningococcal vaccine. 71.2 % had received the mandatory meningococcal quadrivalent vaccine. 74.4% had received their vaccine from hospitals, 13.7% from private clinics, 3.9% from pharmacies, 3.4% from mosques and 4.5% from other places. Figure 1 illustrates the vaccination status of pilgrims by country classification by income.

Approximately one‐third of all vaccinated participants 35.7% received their vaccines at least 2 months before the Hajj. More than half of the participants (55.8%) (49.4–62) said that they took antibiotics during and after the Hajj (Table 2).

**Table 2:** Pilgrim's social and vaccination profile

Variable	Total percentage %
Vaccination status	
Unvaccinated without a vaccination card (self‐reported).	12.6
Unvaccinated with a fake vaccination card (self‐reported).	11
Bivalent (A and C) vaccine.	0.5
Quadrivalent polysaccharide vaccine	54.7
Quadrivalent conjugate vaccine.	16
Unknown meningococcal vaccine.	6.2
Time of vaccination before arrival in Saudi Arabia	
≤60 days	64.3
≥61 days	35.7
Antibiotic use	
Yes	55.8
No	44.2
Smoking status	
Smoker	10.6
Non‐smoker	89.4
Number of pilgrims inside a tent	
6–8	16.4
10–20	13.9
50–100	39.5
≥100	30.3

### Meningococcal carriage rate

A total 37% of the 2249 swabs were positive for *ctrA*, *SodC* and *porA* genes. Target *ctrA* and *porA* genes characteristic of *N. meningitidis* were found among 103 of all 2249 samples tested, giving an overall carriage prevalence of 4.6%. (95% CI: 3.4%–6%); 34 (1.13%) of these samples was positive for serogroups responsible for disease ‐ serogroup A (*n* = 2), B (*n* = 10), C (*n* = 10), W (*n* = 3), X (*n* = 6) and Y (*n* = 3). Both *N. meningitidis* B and C were identified in seven pilgrims, A and B in one, and C and X in one.

### Logistic regression analysis

The logistic analysis was restricted to the 1736 pilgrims who received the meningococcal vaccine and provided an oropharyngeal swab. None of the variables studied was found to be associated with the outcome of carriage at 10% significant level. Meningococcal carriage of serogroup A, C, W and Y *N. meningitidis* was higher among those who received a vaccine more than 60 days before the Hajj than among those who received it 60 days or less before the Hajj, but the difference between groups was not statistically significant (adjusted OR 1.6; 0.55–4.8, *P*‐value 0·36) (Table 3). No other statistically risk factors for *N. meningitidis* carriage were found.

**Table 3:** Risk factors for carriage of *N. meningitidis* ACWY among pilgrims to Mecca, 2017

Exposure	Carriage of *N. meningitides* ACWY			
	Crude odds ratio (95% CI)	*P*‐value	Adjusted odds ratio† (95% CI)	*P*‐value
Time of meningococcal vaccination				
≤60 days	Reference			
≥61	1 (0.34, 3.26)	0.92	1.6 (0.55–4.8)	0.36
Type of meningococcal vaccine				
Bivalent\quadrivalent polysaccharide	Reference			0.67‡
Quadrivalent conjugate	0.73 (0.16, 3.27)	0.8	0.8 (0.08–7.7)	0.8
Unknown	0.37 (0.05, 2.77)	0.33	0.4 (0.05, 4.3)	0.5
Sex				
Female	Reference			
Male	0.72 (0.26–2)	0.53	0.6 (0.22–1.4)	0.25
Age in years				
≤34	Reference			0.81‡
35–44	0.77 (0.11–5.11)	0.78	0.44 (0.04–4.2)	0.4
45–54	0.95 (0.14–6.1)	0.95	0.7(0.08–6)	0.7
55–64	1.8 (0.32–10.5)	0.47	1.(0.16–6.4)	0.9
≥65	1.6 (0.31–8.3)	0.55	1.1 (0.25–5.4)	0.8
Education level				
Low	Reference			0.83‡
Middle	1.(0.20–5.6)	0.93	0.77 (0.15 – 4)	0.7
High	0.5 (0.08–2.9)	0.44	0.53 (0.08–3.6)	0.5
Country classification by income				
Low/lower middle‐income	Reference			0.97‡
Upper middle‐income	0.89 (0.24–3.2)	0.85	1.1 (0.32, 4·1)	0.8
High income	0.54 (0.06–4.7)	0.57	2.3 (0.04, 128)	0.6
Smoking status				
Non‐smoker	Reference			
Smoker	1.3 (0.25–6.5)	0.74	1 (0.10–10)	0.9
Antibiotic use				
No	Reference			
Yes	1.2 (0.38–3.8)	0.72	1 (0.33–3.1)	0.9
Marital status				
Married	Reference			
Unmarried	0.57(0.07–4.2)	0.58	0.57 (0.07–4.4)	0.5
Length of stay in KSA	1 (0.9–1)	0.24	1 (0.9–1)	0.12
Number of pilgrims inside the tent				
6–8	Reference			0.82‡
10–20	0.66 (0.05–8.3)	0.74	0.6(0.05–8.4)	0.7
50–100	0.74 (0.10–5)	0.76	0.8 (0.11, 6)	0.8
≥100	1.5 (0.29–7.6)	0.61	1.4 (0.3, 7.3)	0.6

† The model was adjusted for variables (age, sex, education, type of meningococcal vaccine, timing of meningococcal vaccination, smoking status, marital status, antibiotic use, length of stay in KSA, country classification by income and number of pilgrims inside the tents).

‡ Overall Wald test.

## Discussion

According to the Hajj and Umrah vaccination policy, all pilgrims should receive the mandatory meningococcal quadrivalent (ACYW) vaccine at least 10 days before the Hajj [8, 22]. Pilgrims are also required to submit a certificate to prove that they have received the vaccine [22]. Surprisingly, a quarter of the pilgrims (22·6 %) in this study self‐reported not having been vaccinated against meningococcal disease and of those 10.1 % were carrying unofficial vaccination certificates. The finding of a high percentage of pilgrims being unvaccinated was unexpected and indicates the need to determine whether pilgrims have been vaccinated prior to arrival for Hajj pilgrimage to prevent any future meningococcal outbreaks. An immediate strengthening of the visa/vaccination regulations at Hajj is necessary. The development of a Harmonised Hajj Health Information System (HHIS), a synchronised information and data sharing platform among all Hajj stakeholders, would be beneficial [23]. The HHIS would capture pre‐Hajj data, for instance pilgrims' demographic information, pre‐existing health conditions and vaccination status [23]. To ensure that all pilgrims are receiving mandatory vaccines for the Hajj, vaccines could be given in specific vaccination centres authorised by the Saudi embassies in each country where pilgrims originate from, and the vaccination status is linked electronically to the HHIS.

Our study identified there were more carriers among those vaccinated more than 2 months prior to travelling to the Hajj than among those vaccinated <2 months before arrival, although the difference between groups was not statistically significant. Little information was found in the literature on the question of the association between time of meningococcal vaccination and carriage of *N. meningitidis*. Read et al. conducted the only published study that has investigated this association with regards to meningococcal conjugate vaccine and showed that the natural elimination of existing carriage may take 2 months or more and that the conjugate vaccine can only prevent acquisition of new carriage [9].

There are a number of possible reasons for our findings. Firstly, the number of carriers was relatively small with only 18 being positive for a meningococcus of pathogenic serogroups covered by current vaccines; A,C,W and Y; therefore, a larger study might have found a significant effect. Secondly, over 50% of the pilgrims had received a polysaccharide vaccine, and polysaccharide vaccines have little or no impact on carriage [24]; only 16% were known to have received the quadrivalent conjugate vaccine which has been shown to prevent new acquisition of carriage. The Saudi Hajj vaccination policy does not indicate the type of quadrivalent vaccine required and leaves it to pilgrims to decide [8].

As many pilgrims come from developing countries, the cost of the conjugate vaccine could influence uptake for many [8]. Pilgrims from developing countries make life‐long savings to be able to travel for the Hajj [25] and for many the option of receiving a cheaper polysaccharide vaccine is one that is more appropriate for their financial status. Finally, although the analysis took into account reported use of antibiotics, it is possible that unreported use of antibiotics, which was likely widespread, might have confounded the difference between the groups. The frequent use of antibiotics reported in our study is consistent with other research which found that over 60% of pilgrims who travelled to Saudi Arabia carried antibiotics from their homeland with them and that 39.2% acquired non‐prescribed antibiotics in Saudi Arabia [26]. Other studies have reported misuse and overuse of antibiotics among pilgrims [27] which, if continued, will make Hajj pilgrimage at risk of spreading antibiotic resistance [28].

The overall prevalence of meningococcal carriage in our study was low (4.6%), and that of serogroups A, B, C, W, X and Y that cause the meningococcal disease was very low (1.13%) with many meningococci being non‐groupable. This finding is in agreement with the findings of Memish et al. who also found a low prevalence of carriage of *N. meningitidis* in pilgrims attending the 2014 Hajj [29]. An unexpected finding was the high proportion of carriers carrying meningococci of more than one serogroup. Carrying more than one serogroup of *N*.*meningitidis* in the throat is rare but can occasionally happen [30]. Contrary to expectations, we did not find any significant association between *N. meningitidis (*serogroupable and non‐serogroupable) carriage and any of the risk factors found in other studies (age, sex, education level, smoking status, marital status, type of meningococcal vaccination, timing of vaccination, country classification by income and number of pilgrims inside the tents) [31]. Detection of serogroup X among the pilgrims is of concern as *N. meningitidis* serogroup X has the potential to cause epidemics, as experienced recently in the African meningitis belt [32]. Currently, there is no licensed vaccine against serogroup X *N. meningitides* [33] although a pentavalent conjugate vaccine containing a serogroup X conjugate is being developed by the Serum Institute of India and is undergoing clinical trials [34].

This study had a number of limitations. Only pilgrims that had completed the Hajj were included, and there was a lack of information on events before the Hajj, for example, those who attended a Hajj camp before the pilgrimage. Our findings are limited by the use of a cross‐sectional design, given that conducting a large‐scale longitudinal study is challenging and costly in circumstances similar to the Hajj. In addition, information on antibiotic use was self‐reported, and some pilgrims may have confused use of antibiotics with other non‐antibiotic medications. The percentage of those unvaccinated is limited to those who have reported not being vaccinated, and the number could be larger than recognised. Despite these limitations, this study was one of the largest studies undertaken on meningococcal carriage in pilgrims after completing the Hajj. Results have raised many questions regarding the need for further investigation including the issue of pilgrims travelling with unofficially purchased vaccination cards. Our study determined the prevalence of unvaccinated pilgrims and those with unofficial vaccination cards, but the reasons behind this remain to be explored. Pilgrims from different countries could potentially have different reasons. A follow‐up qualitative phase of the study may provide information to explore the issue of unofficial vaccination cards more comprehensively.

One of the primary reasons for the authorities' policy on meningococcal vaccination prior to the Hajj is to prevent spread of meningococci among pilgrims during the Hajj, as well as to protect them against invasive meningococcal disease. More studies are needed to ascertain the importance of using a conjugate rather than a polysaccharide vaccine and on the optimum vaccination time prior to travelling to the Hajj.

## References

[1] Rosenstein NE , Perkins BA , Stephens DS , Popovic T , Hughes JM . Meningococcal disease. N Eng J Med 2001: 344(18): 1378–1388. 10.1056/NEJM200105033441807 11333996

[2] Pollard AJ . Global epidemiology of meningococcal disease and vaccine efficacy. Pediatr Infect Dis J. 2004: 23(12 Suppl): S274–S279. 15597069

[3] Stephens DS . Uncloaking the meningococcus: dynamics of carriage and disease. Lancet. 1999: 353(9157): 941–942. 10459897 10.1016/S0140-6736(98)00279-7

[4] Yezli S , Saeed AAB , Assiri AM *et al*. Prevention of meningococcal disease during the Hajj and Umrah mass gatherings: past and current measures and future prospects. Int J Infect Dis 2016: 47: 71–78. 26707071 10.1016/j.ijid.2015.12.010

[5] Imam A , Alamoudi M , editors. Mina: the city of tents origination and development. 9° Congresso Città e Territorio Virtuale: Roma, 2, 3 e 4 ottobre 2013; 2014: Università degli Studi Roma Tre.

[6] Memish Z , Al Hakeem R , Al Neel O , Danis K , Jasir A , Eibach D . Laboratory‐confirmed invasive meningococcal disease: effect of the Hajj vaccination policy. Saudi Arabia, 1995 to 2011. Euro surveillance 2013;18(37):20581. 24079399 10.2807/1560-7917.es2013.18.37.20581

[7] Crum‐Cianflone N , Sullivan E . Meningococcal vaccinations. Infect Dis Ther 2016: 5(2): 89–112. 27086142 10.1007/s40121-016-0107-0 PMC4929086

[8] 1441H.‐Hajj Season [Internet] . Saudi ministry of health: Saudi Arabia Accessed February 8, 2020; 2020 [cited 2020]. (Available from: https://www.moh.gov.sa/en/Hajj/Pages/hajj2019.aspx

[9] Read RC , Baxter D , Chadwick DR *et al*. Effect of a quadrivalent meningococcal ACWY glycoconjugate or a serogroup B meningococcal vaccine on meningococcal carriage: an observer‐blind, phase 3 randomised clinical trial. Lancet 2014: 384(9960): 2123–2131. 25145775 10.1016/S0140-6736(14)60842-4

[10] Rashid H , Khatami A , Haworth E , Booy R . Meningococcal vaccination and Hajj pilgrimage. Lancet 2015: 385(9973): 1072–1073. 10.1016/S0140-6736(15)60598-0 25797556

[11] Balkhy HH , Memish ZA , Almuneef MA , Osoba AO . Neisseria meningitidis W‐135 carriage during the Hajj season 2003. Scand J Infect Dis 2004: 36(4): 264–268. 15198182 10.1080/00365540410020082

[12] Husain EH , Dashti AA , Electricwala QY , AbdulSamad AM , Al‐Sayegh S . Absence of Neisseria meningitidis from throat swabs of Kuwaiti pilgrims after returning from the Hajj. Med Prin Pract 2010: 19(4): 321–323. 10.1159/000312721 20516711

[13] Alborzi A , Oskoee S , Pourabbas B *et al*. Meningococcal carrier rate before and after hajj pilgrimage: effect of single dose ciprofloxacin on carriage. East Mediterr Health J 2008: 14: 277–282. 18561718

[14] Memish ZA , Yezli S , Almasri M *et al*. Meningococcal serogroup A, C, W, and Y serum bactericidal antibody profiles in Hajj pilgrims. Int J Infect Dis 2014: 28: 171–175. 25307887 10.1016/j.ijid.2014.09.005

[15] LSHTM Open Data Kit [Internet]. London School of Hygiene & Tropical Medicine; 2019. (Available from: https://opendatakit.lshtm.ac.uk/) [17 Mar 2019]

[16] Jordens JZ , Heckels JE . A novel porA‐based real‐time PCR for detection of meningococcal carriage. J Med Microbiol 2005: 54(5): 463–466. 15824424 10.1099/jmm.0.45847-0

[17] CDC . Chapter 10 Popular Itineraries Africa & the Middle East [Internet]. Accessed February 8, 2020: 2019. (Available from: https://wwwnc.cdc.gov/travel/yellowbook/2020/popular‐itineraries/saudi‐arabia‐hajjumrah‐pilgrimage

[18] Rojas E , Hoyos J , Oldfield NJ *et al*. Optimization of molecular approaches to genogroup Neisseria meningitidis carriage isolates and implications for monitoring the impact of new serogroup B vaccines. PLoS One 2015: 10(7): e0132140. 26147212 10.1371/journal.pone.0132140 PMC4493136

[19] Sacchi CT , Fukasawa LO , Gonçalves MG *et al*. Incorporation of real‐time PCR into routine public health surveillance of culture negative bacterial meningitis in São Paulo, Brazil. PLoS One 2011: 6(6): e20675. 21731621 10.1371/journal.pone.0020675 PMC3120771

[20] New country classifications by income level: 2017–2018 [Internet]. World Bank Bloges 2017–2018. (Available from: https://blogs.worldbank.org/opendata/new‐country‐classifications‐income‐level‐2017‐2018) [2 Jul 2019]

[21] StataCorp . StataCorp. Stata Statistical Software: Release 16. College Station, TX: StataCorp LLC [Internet]. Accessed May 20, 2019: 2019. (Available from: https://www.stata.com/

[22] MFA . Hajj Requirements [Internet]. Accessed May 20, 2019: Washington DC: Royal Embassy of Saudi Arabia 2019. (Available from: https://www.saudiembassy.net/hajj‐requirements

[23] Yezli S , Elganainy A , Awam A . Strengthening health security at the Hajj mass gatherings: a Harmonised Hajj health information system. J Trav Med 2018; 25(1): tay070. 10.1093/jtm/tay070 30137404

[24] Poland GA . Prevention of meningococcal disease: current use of polysaccharide and conjugate vaccines. Clin Infect Dis 2010: 50(s2): S45–S53. 20144016 10.1086/648964

[25] Memish ZA , Elachola H , Rahman M , Sow S , Aljerian N , Assiri A . Objection to chronic disease based restrictions during the Hajj. Lancet 2016: 387(10029): 1719. 10.1016/S0140-6736(16)30257-4 27116275

[26] Yezli S , Yassin Y , Mushi A *et al*. Knowledge, attitude and practice (KAP) survey regarding antibiotic use among pilgrims attending the 2015 Hajj mass gathering. Trav Med Infect Dis 2019: 28: 52–58. 10.1016/j.tmaid.2018.08.004 30118860

[27] Azeem M , Tashani M , Barasheed O *et al*. Knowledge, attitude and practice (KAP) survey concerning antimicrobial use among Australian Hajj Pilgrims. Infect Disord Drug Targets 2014: 14(2): 125–132. 25019233 10.2174/1871526514666140713161757

[28] Al‐Tawfiq JA , Memish ZA . Potential risk for drug resistance globalization at the Hajj. Clin Microbiol Infect 2015: 21(2): 109–114. 25682276 10.1016/j.cmi.2014.11.013

[29] Memish ZA , Al‐Tawfiq JA , Almasri M *et al*. Neisseria meningitidis nasopharyngeal carriage during the Hajj: a cohort study evaluating the need for ciprofloxacin prophylaxis. Vaccine 2017: 35(18): 2473–2478. 28343777 10.1016/j.vaccine.2017.03.027

[30] Caugant DA , Tzanakaki G , Kriz P . Lessons from meningococcal carriage studies. FEMS Microbiol Rev 2007: 31(1): 52–63. 17233635 10.1111/j.1574-6976.2006.00052.x

[31] Caugant DA , Maiden MC . Meningococcal carriage and disease–population biology and evolution. Vaccine 2009: 24(27 Suppl 2): B64–B70. 10.1016/j.vaccine.2009.04.061 PMC2719693 19464092

[32] Delrieu I , Yaro S , Tamekloe TA *et al*. Emergence of epidemic Neisseria meningitidis serogroup X meningitis in Togo and Burkina Faso. PLoS One 2011: 6(5): e19513. 21625480 10.1371/journal.pone.0019513 PMC3098835

[33] Piccini G , Torelli A , Gianchecchi E , Piccirella S , Montomoli E . Fighting Neisseria meningitidis: past and current vaccination strategies. Exp Rev Vac 2016: 15(11): 1393–1407. 10.1080/14760584.2016.1187068 27158988

[34] Chen WH , Neuzil KM , Boyce CR *et al*. Safety and immunogenicity of a pentavalent meningococcal conjugate vaccine containing serogroups A, C, Y, W, and X in healthy adults: a phase 1, single‐centre, double‐blind, randomised, controlled study. Lancet Infect Dis 2018: 18(10): 1088–1096. 30120069 10.1016/S1473-3099(18)30400-6

